# 5,5′-(Ethyne-1,2-di­yl)diisophthalic acid dimethyl sulfoxide tetra­solvate

**DOI:** 10.1107/S1600536813013068

**Published:** 2013-05-18

**Authors:** Alexander S. Münch, Felix Katzsch, Edwin Weber, Florian O. R. L. Mertens

**Affiliations:** a Institute of Physical Chemistry, Technical University Bergakademie Freiberg, Leipziger Strasse 29, 09596 Freiberg/Sachsen, Germany; b Institute of Organic Chemistry, Technical University Bergakademie Freiberg, Leipziger Strasse 29, 09596 Freiberg/Sachsen, Germany

## Abstract

In the title compound, C_18_H_10_O_8_·4C_2_H_6_OS, the mid-point of the triple bond of the main mol­ecule is located on a special position, *i.e.* about an inversion center. The carboxyl groups are twisted slightly out of the planes of the aromatic rings to which they are attached, making dihedral angles of 24.89 (1) and 7.40 (2)°. The cystal packing features strong O—H⋯O hydrogen bonds, weaker C—H⋯O inter­actions and O⋯S contacts [3.0981 (11) Å] and displays channel-like voids extending along the *a-*axis direction which contain the dimethyl sulfoxide solvent mol­ecules.

## Related literature

For the synthesis of the principal compound, see: Hausdorf *et al.* (2009 ▶); Zhou *et al.* (2007 ▶). For its use as linker mol­ecule in the formation of porous metal–organic framework structures, see: Hausdorf *et al.* (2009 ▶); Hu *et al.* (2009 ▶); Zheng *et al.* (2013 ▶). For metal–organic frameworks, see: Münch *et al.* (2011 ▶); Chen *et al.* (2005 ▶); Coles *et al.* (2002 ▶). For a similar hydrogen-bonded aggregate, see: Hauptvogel *et al.* (2011 ▶). For O—H⋯O hydrogen bonds, see: Bernstein *et al.* (1995 ▶); Katzsch *et al.* (2011 ▶). For C—H⋯O contacts, see: Desiraju & Steiner (1999 ▶); Katzsch & Weber (2012 ▶); Fischer *et al.* (2011 ▶). For O⋯S contacts, see: Lu *et al.* (2011 ▶). For π–π inter­actions, see: Hunter & Sanders (1990 ▶).
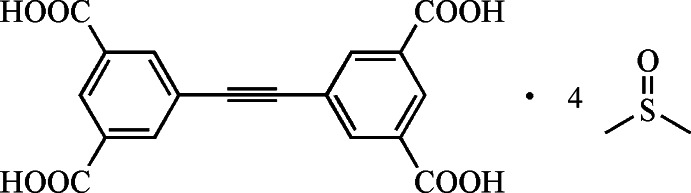


## Experimental

### 

#### Crystal data

C_18_H_10_O_8_·4C_2_H_6_OS
*M* *_r_* = 666.81
Monoclinic, 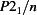

*a* = 8.1406 (2) Å
*b* = 8.7328 (2) Å
*c* = 21.4351 (5) Å
β = 95.970 (1)°
*V* = 1515.56 (6) Å^3^
*Z* = 2
Mo *K*α radiation
μ = 0.38 mm^−1^
*T* = 100 K
0.60 × 0.42 × 0.36 mm

#### Data collection

Bruker APEXII CCD diffractometer
Absorption correction: multi-scan (*SADABS*; Sheldrick, 2004 ▶) *T*_min_ = 0.807, *T*_max_ = 0.877
20635 measured reflections
2666 independent reflections
2567 reflections with *I* > 2σ(*I*)
*R*_int_ = 0.021

#### Refinement

*R*[*F* ^2^ > 2σ(*F* ^2^)] = 0.024
*wR*(*F* ^2^) = 0.059
*S* = 1.04
2666 reflections
197 parameters
H-atom parameters constrained
Δρ_max_ = 0.32 e Å^−3^
Δρ_min_ = −0.28 e Å^−3^

### 

Data collection: *APEX2* (Bruker, 2007 ▶); cell refinement: *SAINT* (Bruker, 2007 ▶); data reduction: *SAINT*; program(s) used to solve structure: *SHELXS97* (Sheldrick, 2008 ▶); program(s) used to refine structure: *SHELXL97* (Sheldrick, 2008 ▶); molecular graphics: *SHELXTL* (Sheldrick, 2008 ▶); software used to prepare material for publication: *SHELXTL*.

## Supplementary Material

Crystal structure: contains datablock(s) I, global. DOI: 10.1107/S1600536813013068/rk2402sup1.cif

Structure factors: contains datablock(s) I. DOI: 10.1107/S1600536813013068/rk2402Isup2.hkl

Supplementary material file. DOI: 10.1107/S1600536813013068/rk2402Isup3.cml

Additional supplementary materials:  crystallographic information; 3D view; checkCIF report

## Figures and Tables

**Table 1:** Hydrogen-bond geometry (Å, °)

*D*—H⋯*A*	*D*—H	H⋯*A*	*D*⋯*A*	*D*—H⋯*A*
O1—H1⋯O1*G* ^i^	0.84	1.71	2.5451 (13)	171
O3—H3⋯O1*H* ^ii^	0.84	1.76	2.5732 (13)	161
C1*G*—H1*G*2⋯O2^iii^	0.98	2.56	3.3138 (17)	134
C1*G*—H1*G*3⋯O4^iv^	0.98	2.71	3.5351 (17)	143
C2*G*—H2*G*1⋯O1*H* ^v^	0.98	2.57	3.5093 (18)	160
C2*G*—H2*G*2⋯O2^iii^	0.98	2.52	3.2783 (17)	135
C1*H*—H1*H*1⋯O4^vi^	0.98	2.57	3.5006 (18)	159
C1*H*—H1*H*2⋯O4^vii^	0.98	2.69	3.4427 (18)	134
C2*H*—H2*H*1⋯O1*H* ^v^	0.98	2.52	3.3409 (17)	141
C2*H*—H2*H*2⋯O1^viii^	0.98	2.67	3.4738 (17)	139
C2*H*—H2*H*2⋯O1*G* ^ix^	0.98	2.54	3.1574 (17)	121
C2*H*—H2*H*2⋯O4^vii^	0.98	2.70	3.4604 (18)	135

Symmetry codes: (i) 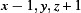; (ii) 
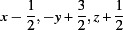
; (iii) 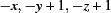; (iv) 
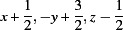
; (v) 
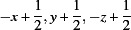
; (vi) 
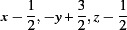
; (vii) 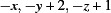; (viii) 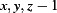; (ix) 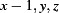.

**Fig. 1.**
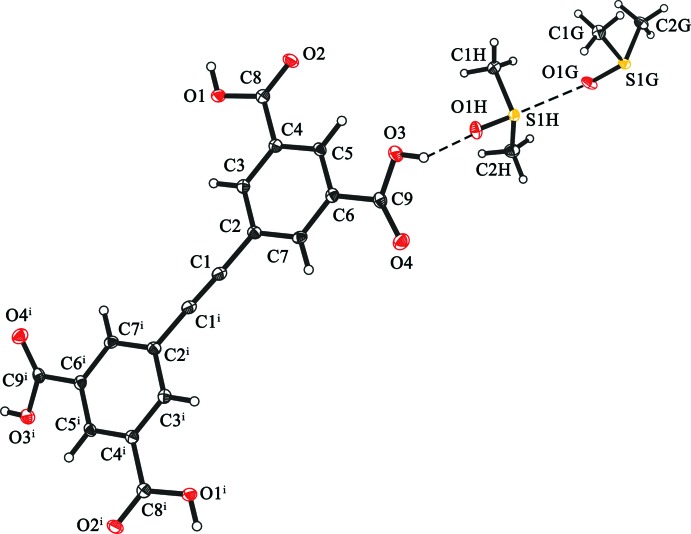
Perspective view of the title compound, including atom numbering scheme. Displacement ellipsoids are drawn at 50% probability level. H atoms are presented as a small spheres of arbitrary radius.

**Fig. 2.**
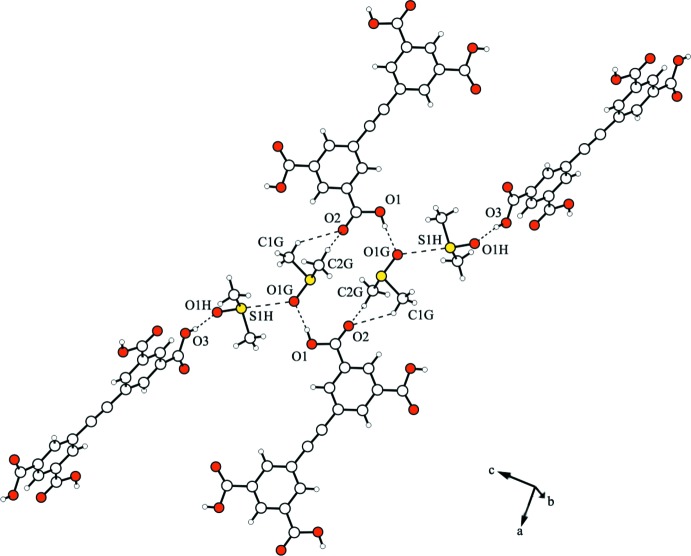
Selected intermolecular interactions within the layer structure of the solvate.

**Fig. 3.**
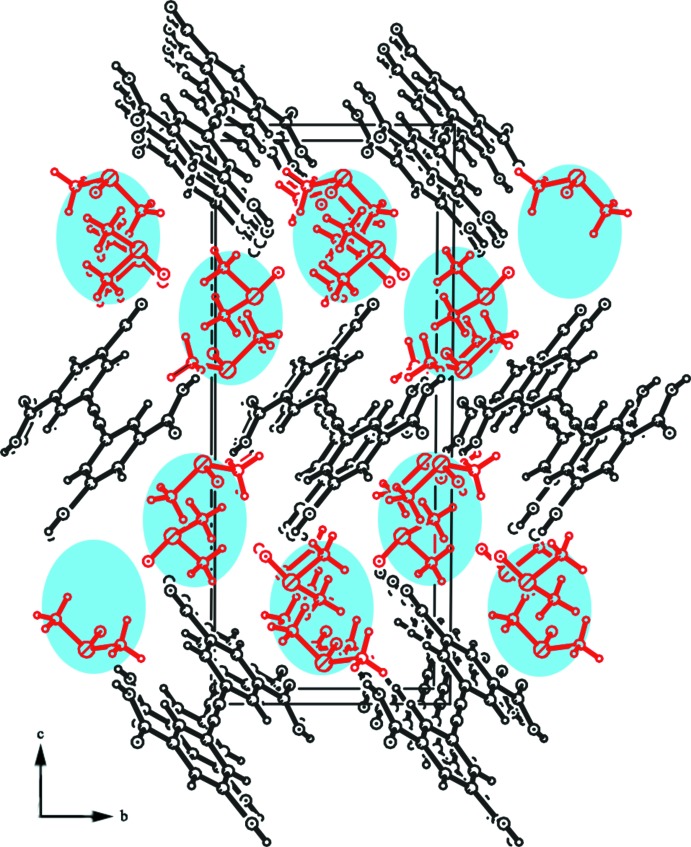
Solvent channels along the crystallographic a–axis in the packing structure.

## supplementary crystallographic information

### Comment

During the last years tetracarboxylic acid linker molecules of which 3,3',5,5'-biphenyltetracarboxylic acid is the prototype have proven highly effective both in the construction of porous metal–organic (MOF) (Chen *et al.*, 2005; Münch *et al.*, 2011) and hydrogen bond supported frameworks (Coles *et al.*, 2002) as well as in the formation of hydrogen bond assembled layer structures (Zhou *et al.*, 2007). Insertion of an ethynylene unit into the molecular backbone such as in the title compound, 5,5'–(ethynylene)diisophthalic acid, was undertaken in order to expand lattice porosity and also to introduce an additional interaction site for improved solid–gas adsorption behaviour (Hausdorf *et al.*, 2009; Zheng *et al.*, 2013). This has been confirmed showing high acetylene uptake of a corresponding MOF-framework (Hu *et al.*, 2009). But as a rigid tetrafunctional carboxylic acid, the title compound should also capable of forming complex hydrogen bonded aggregate structures in the solid state (Hauptvogel *et al.*, 2011) of which the present solvate with dimethyl sulfoxide finishes another evident proof. The title compound crystallizes in the monoclinic space group *P*2_1_/*n* with half a molecule of 5,5'-(ethynylene)diisophthalic acid and two dimethyl sulfoxide molecules in the asymmetric part of the unit cell. The tolane fragment devites from ideal linear geometry (C2—C1≡C1^i^ = 178.29 (18)°) and the carboxyl groups are slightly twisted out of the aromatic ring plane - dihedral angles 24.89 (1)° (O4═C9—O3) and 7.40 (2)° (O2═C8—O1)]. The principal molecules are vertically oriented to each other in a layer structure connected by two consecutively arranged solvent molecules *via* strong O—H···O hydrogen bonds (Bernstein *et al.*, 1995; Katzsch *et al.*, 2011) [*d*(O1···O1G^i^) = 2.55Å, *d*(O3···O1H^ii^) = 2.57Å], O···S contacts [*d*(O1G···S1H) = 3.10Å] (Lu *et al.*, 2011) as well as weak C—H···O interactions [*d*(C1G···O2^iii^) = 3.31Å, *d*(C2G···O2^iii^)= 3.28Å and *d*(C2G···O1H^v^) = 3.51Å] (Desiraju & Steiner, 1999; Katzsch & Weber, 2012; Fischer *et al.*, 2011). Superimposed tapes are held together by π-π interactions between the aromatic rings (Hunter & Sanders, 1990) and the interacting solvent molecules being included in channels along the crystallopgraphic *a*–axis. Symmetry code: (i) -*x*+1, -*y*+2, -*z*+2.

### Experimental

The titled compound was synthesized *via* a Sonogashira–Hagihara cross coupling reaction of dimethyl 5-ethynylisophthalate and dimethyl 5-iodoisophthalate. For the synthetic procedure, see: Hausdorf *et al.* (2009), Zhou *et al.* (2007). Colourless single crystals suitable for *X*-ray diffraction were grown by slow evaporation from a dimethyl sulfoxide/mesitylene (2:1) solution.

### Refinement

The H atoms were positioned geometrically and allowed to ride on their parent atoms, with O—H = 0.84Å and *U*_iso_(H) = 1.5*U*_eq_(O) for hydroxyl H atoms, C—H = 0.95Å and *U*_iso_(H) = 1.2*U*_eq_(C) for aryl H atoms, and C—H = 0.98Å and *U*_iso_(H) = 1.5*U*_eq_(C) for methyl H atoms.

### Crystal data

**Table:**

C_18_H_10_O_8_·4C_2_H_6_OS	*F*(000) = 700
*M**_r_* = 666.81	*D*_x_ = 1.461 Mg m^−^^3^
Monoclinic, *P*2_1_/*n*	Melting point > 623 K
Hall symbol: -P 2yn	Mo *K*α radiation, λ = 0.71073 Å
*a* = 8.1406 (2) Å	Cell parameters from 9934 reflections
*b* = 8.7328 (2) Å	θ = 2.5–49.6°
*c* = 21.4351 (5) Å	µ = 0.38 mm^−^^1^
β = 95.970 (1)°	*T* = 100 K
*V* = 1515.56 (6) Å^3^	Block, colourless
*Z* = 2	0.60 × 0.42 × 0.36 mm

### Data collection

**Table:**

Bruker APEXII CCD diffractometer	2666 independent reflections
Radiation source: fine-focus sealed tube	2567 reflections with *I* > 2σ(*I*)
Graphite monochromator	*R*_int_ = 0.021
ω– and φ–scans	θ_max_ = 25.0°, θ_min_ = 2.5°
Absorption correction: multi-scan (*SADABS*; Sheldrick, 2004)	*h* = −9→9
*T*_min_ = 0.807, *T*_max_ = 0.877	*k* = −10→10
20635 measured reflections	*l* = −25→25

### Refinement

**Table:**

Refinement on F^2^	Secondary atom site location: difference Fourier map
Least-squares matrix: full	Hydrogen site location: inferred from neighbouring sites
R[F^2^ > 2σ(F^2^)] = 0.024	H-atom parameters constrained
wR(F^2^) = 0.059	w = 1/[σ^2^(F_o_^2^) + (0.0228P)^2^ + 1.1796P] where P = (F_o_^2^ + 2F_c_^2^)/3
S = 1.04	(Δ/σ)_max_ = 0.001
2666 reflections	Δρ_max_ = 0.32 e Å^−^^3^
197 parameters	Δρ_min_ = −0.28 e Å^−^^3^
0 restraints	Extinction correction: SHELXL97 (Sheldrick, 2008), Fc^*^=kFc[1+0.001xFc^2^λ^3^/sin(2θ)]^-1/4^
Primary atom site location: structure-invariant direct methods	Extinction coefficient: 0.0065 (7)

### Special details

**Table:**

Geometry. All s.u.'s (except the s.u. in the dihedral angle between two l.s. planes) are estimated using the full covariance matrix. The cell s.u.'s are taken into account individually in the estimation of s.u.'s in distances, angles and torsion angles; correlations between s.u.'s in cell parameters are only used when they are defined by crystal symmetry. An approximate (isotropic) treatment of cell s.u.'s is used for estimating s.u.'s involving l.s. planes.
Refinement. Refinement of *F*^2^ against ALL reflections. The weighted *R*–factor *wR* and goodness of fit *S* are based on *F*^2^, conventional *R*–factors *R* are based on *F*, with *F* set to zero for negative *F*^2^. The threshold expression of *F*^2^ > 2σ(*F*^2^) is used only for calculating *R*–factors(gt) *etc*. and is not relevant to the choice of reflections for refinement. *R*–factors based on *F*^2^ are statistically about twice as large as those based on *F*, and *R*–factors based on ALL data will be even larger.

### Fractional atomic coordinates and isotropic or equivalent isotropic displacement parameters (Å^2^)

**Table:**

	*x*	*y*	*z*	*U*_iso_*/*U*_eq_	
O1	−0.01957 (11)	0.64073 (11)	1.04506 (4)	0.0150 (2)	
H1	−0.0882	0.5906	1.0635	0.023*	
O2	−0.23612 (11)	0.67476 (11)	0.97291 (5)	0.0171 (2)	
O3	−0.21203 (11)	1.12870 (12)	0.83853 (4)	0.0175 (2)	
H3	−0.2524	1.1864	0.8095	0.026*	
O4	0.02782 (12)	1.18846 (11)	0.80107 (5)	0.0188 (2)	
C1	0.43140 (17)	0.98735 (16)	0.98804 (6)	0.0140 (3)	
C2	0.26574 (16)	0.95657 (15)	0.96125 (6)	0.0126 (3)	
C3	0.17287 (16)	0.84319 (15)	0.98761 (6)	0.0124 (3)	
H3A	0.2215	0.7840	1.0219	0.015*	
C4	0.01012 (16)	0.81681 (15)	0.96390 (6)	0.0120 (3)	
C5	−0.06279 (16)	0.90560 (15)	0.91469 (6)	0.0122 (3)	
H5	−0.1751	0.8894	0.8993	0.015*	
C6	0.02860 (16)	1.01785 (15)	0.88804 (6)	0.0123 (3)	
C7	0.19281 (16)	1.04245 (15)	0.91065 (6)	0.0128 (3)	
H7	0.2556	1.1178	0.8917	0.015*	
C8	−0.09463 (16)	0.70282 (15)	0.99368 (6)	0.0127 (3)	
C9	−0.04995 (16)	1.12030 (15)	0.83737 (6)	0.0136 (3)	
O1G	0.78072 (12)	0.50559 (12)	0.11049 (5)	0.0203 (2)	
S1G	0.61418 (4)	0.44680 (4)	0.081415 (15)	0.01417 (10)	
C1G	0.55281 (17)	0.31280 (16)	0.13740 (7)	0.0179 (3)	
H1G1	0.6343	0.2300	0.1430	0.027*	
H1G2	0.4446	0.2701	0.1224	0.027*	
H1G3	0.5459	0.3647	0.1776	0.027*	
C2G	0.47196 (17)	0.59699 (16)	0.09296 (7)	0.0187 (3)	
H2G1	0.4814	0.6252	0.1374	0.028*	
H2G2	0.3592	0.5620	0.0799	0.028*	
H2G3	0.4971	0.6862	0.0679	0.028*	
O1H	0.10288 (12)	0.21753 (11)	0.25639 (5)	0.0197 (2)	
S1H	−0.00487 (4)	0.32301 (4)	0.212754 (15)	0.01365 (10)	
C1H	−0.09563 (18)	0.45741 (18)	0.26201 (7)	0.0212 (3)	
H1H1	−0.1821	0.4065	0.2829	0.032*	
H1H2	−0.1439	0.5424	0.2365	0.032*	
H1H3	−0.0105	0.4969	0.2936	0.032*	
C2H	0.13616 (17)	0.45062 (17)	0.18084 (7)	0.0181 (3)	
H2H1	0.2159	0.4890	0.2146	0.027*	
H2H2	0.0750	0.5368	0.1604	0.027*	
H2H3	0.1949	0.3960	0.1500	0.027*	

### Atomic displacement parameters (Å^2^)

**Table:**

	*U*^11^	*U*^22^	*U*^33^	*U*^12^	*U*^13^	*U*^23^
O1	0.0133 (5)	0.0160 (5)	0.0157 (5)	−0.0021 (4)	0.0013 (4)	0.0042 (4)
O2	0.0125 (5)	0.0181 (5)	0.0201 (5)	−0.0040 (4)	−0.0010 (4)	0.0017 (4)
O3	0.0128 (5)	0.0213 (5)	0.0175 (5)	0.0016 (4)	−0.0024 (4)	0.0052 (4)
O4	0.0194 (5)	0.0200 (5)	0.0172 (5)	0.0009 (4)	0.0030 (4)	0.0055 (4)
C1	0.0133 (6)	0.0144 (7)	0.0148 (6)	0.0008 (5)	0.0034 (5)	0.0024 (5)
C2	0.0102 (6)	0.0144 (6)	0.0136 (6)	0.0012 (5)	0.0028 (5)	−0.0030 (5)
C3	0.0125 (6)	0.0129 (6)	0.0119 (6)	0.0025 (5)	0.0014 (5)	−0.0007 (5)
C4	0.0125 (6)	0.0114 (6)	0.0124 (6)	0.0006 (5)	0.0026 (5)	−0.0036 (5)
C5	0.0107 (6)	0.0138 (6)	0.0119 (6)	0.0000 (5)	0.0004 (5)	−0.0039 (5)
C6	0.0134 (6)	0.0128 (6)	0.0110 (6)	0.0017 (5)	0.0019 (5)	−0.0032 (5)
C7	0.0129 (6)	0.0129 (6)	0.0132 (6)	−0.0004 (5)	0.0047 (5)	−0.0018 (5)
C8	0.0138 (7)	0.0109 (6)	0.0135 (6)	0.0015 (5)	0.0023 (5)	−0.0027 (5)
C9	0.0150 (7)	0.0128 (6)	0.0126 (6)	0.0000 (5)	−0.0005 (5)	−0.0033 (5)
O1G	0.0126 (5)	0.0287 (6)	0.0191 (5)	−0.0055 (4)	−0.0007 (4)	0.0071 (4)
S1G	0.01290 (18)	0.01668 (18)	0.01294 (17)	−0.00069 (13)	0.00131 (12)	0.00166 (12)
C1G	0.0165 (7)	0.0184 (7)	0.0188 (7)	−0.0021 (6)	0.0020 (5)	0.0053 (6)
C2G	0.0170 (7)	0.0164 (7)	0.0220 (7)	0.0015 (6)	−0.0001 (5)	0.0003 (6)
O1H	0.0208 (5)	0.0154 (5)	0.0210 (5)	−0.0003 (4)	−0.0066 (4)	0.0022 (4)
S1H	0.01273 (17)	0.01452 (18)	0.01321 (17)	−0.00085 (13)	−0.00095 (12)	−0.00052 (12)
C1H	0.0205 (7)	0.0250 (8)	0.0188 (7)	0.0030 (6)	0.0054 (6)	−0.0024 (6)
C2H	0.0152 (7)	0.0205 (7)	0.0187 (7)	−0.0021 (6)	0.0028 (5)	0.0009 (6)

### Geometric parameters (Å, º)

**Table:**

O1—C8	1.3192 (16)	O1G—S1G	1.5213 (10)
O1—H1	0.8400	S1G—C2G	1.7837 (14)
O2—C8	1.2158 (16)	S1G—C1G	1.7843 (14)
O3—C9	1.3243 (16)	C1G—H1G1	0.9800
O3—H3	0.8400	C1G—H1G2	0.9800
O4—C9	1.2096 (17)	C1G—H1G3	0.9800
C1—C1^i^	1.200 (3)	C2G—H2G1	0.9800
C1—C2	1.4351 (19)	C2G—H2G2	0.9800
C2—C7	1.3990 (19)	C2G—H2G3	0.9800
C2—C3	1.3999 (19)	O1H—S1H	1.5239 (10)
C3—C4	1.3880 (18)	S1H—C2H	1.7864 (14)
C3—H3A	0.9500	S1H—C1H	1.7890 (14)
C4—C5	1.3913 (19)	C1H—H1H1	0.9800
C4—C8	1.4963 (18)	C1H—H1H2	0.9800
C5—C6	1.3890 (19)	C1H—H1H3	0.9800
C5—H5	0.9500	C2H—H2H1	0.9800
C6—C7	1.3904 (19)	C2H—H2H2	0.9800
C6—C9	1.4982 (18)	C2H—H2H3	0.9800
C7—H7	0.9500		
			
C8—O1—H1	109.5	C2G—S1G—C1G	99.07 (7)
C9—O3—H3	109.5	S1G—C1G—H1G1	109.5
C1^i^—C1—C2	178.29 (18)	S1G—C1G—H1G2	109.5
C7—C2—C3	119.25 (12)	H1G1—C1G—H1G2	109.5
C7—C2—C1	121.00 (12)	S1G—C1G—H1G3	109.5
C3—C2—C1	119.70 (12)	H1G1—C1G—H1G3	109.5
C4—C3—C2	120.31 (12)	H1G2—C1G—H1G3	109.5
C4—C3—H3A	119.8	S1G—C2G—H2G1	109.5
C2—C3—H3A	119.8	S1G—C2G—H2G2	109.5
C3—C4—C5	120.05 (12)	H2G1—C2G—H2G2	109.5
C3—C4—C8	121.26 (12)	S1G—C2G—H2G3	109.5
C5—C4—C8	118.49 (12)	H2G1—C2G—H2G3	109.5
C6—C5—C4	120.04 (12)	H2G2—C2G—H2G3	109.5
C6—C5—H5	120.0	O1H—S1H—C2H	105.09 (6)
C4—C5—H5	120.0	O1H—S1H—C1H	106.32 (6)
C5—C6—C7	120.15 (12)	C2H—S1H—C1H	97.93 (7)
C5—C6—C9	120.87 (12)	S1H—C1H—H1H1	109.5
C7—C6—C9	118.87 (12)	S1H—C1H—H1H2	109.5
C6—C7—C2	120.16 (12)	H1H1—C1H—H1H2	109.5
C6—C7—H7	119.9	S1H—C1H—H1H3	109.5
C2—C7—H7	119.9	H1H1—C1H—H1H3	109.5
O2—C8—O1	124.18 (12)	H1H2—C1H—H1H3	109.5
O2—C8—C4	122.56 (12)	S1H—C2H—H2H1	109.5
O1—C8—C4	113.23 (11)	S1H—C2H—H2H2	109.5
O4—C9—O3	125.03 (12)	H2H1—C2H—H2H2	109.5
O4—C9—C6	123.21 (12)	S1H—C2H—H2H3	109.5
O3—C9—C6	111.74 (11)	H2H1—C2H—H2H3	109.5
O1G—S1G—C2G	104.99 (6)	H2H2—C2H—H2H3	109.5
O1G—S1G—C1G	104.22 (6)		
			
C7—C2—C3—C4	−0.23 (19)	C3—C2—C7—C6	1.70 (19)
C1—C2—C3—C4	177.18 (12)	C1—C2—C7—C6	−175.67 (12)
C2—C3—C4—C5	−1.53 (19)	C3—C4—C8—O2	−178.09 (12)
C2—C3—C4—C8	−176.32 (12)	C5—C4—C8—O2	7.04 (19)
C3—C4—C5—C6	1.83 (19)	C3—C4—C8—O1	3.81 (17)
C8—C4—C5—C6	176.76 (11)	C5—C4—C8—O1	−171.06 (11)
C4—C5—C6—C7	−0.36 (19)	C5—C6—C9—O4	−158.78 (13)
C4—C5—C6—C9	−176.70 (12)	C7—C6—C9—O4	24.84 (19)
C5—C6—C7—C2	−1.41 (19)	C5—C6—C9—O3	22.60 (17)
C9—C6—C7—C2	175.00 (12)	C7—C6—C9—O3	−153.78 (12)

Symmetry code: (i) −*x*+1, −*y*+2, −*z*+2.

### Hydrogen-bond geometry (Å, º)

**Table:**

*D*—H···*A*	*D*—H	H···*A*	*D*···*A*	*D*—H···*A*
O1—H1···O1*G*^ii^	0.84	1.71	2.5451 (13)	171
O3—H3···O1*H*^iii^	0.84	1.76	2.5732 (13)	161
C1*G*—H1*G*2···O2^iv^	0.98	2.56	3.3138 (17)	134
C1*G*—H1*G*3···O4^v^	0.98	2.71	3.5351 (17)	143
C2*G*—H2*G*1···O1*H*^vi^	0.98	2.57	3.5093 (18)	160
C2*G*—H2*G*2···O2^iv^	0.98	2.52	3.2783 (17)	135
C1*H*—H1*H*1···O4^vii^	0.98	2.57	3.5006 (18)	159
C1*H*—H1*H*2···O4^viii^	0.98	2.69	3.4427 (18)	134
C2*H*—H2*H*1···O1*H*^vi^	0.98	2.52	3.3409 (17)	141
C2*H*—H2*H*2···O1^ix^	0.98	2.67	3.4738 (17)	139
C2*H*—H2*H*2···O1*G*^x^	0.98	2.54	3.1574 (17)	121
C2*H*—H2*H*2···O4^viii^	0.98	2.70	3.4604 (18)	135

Symmetry codes: (ii) *x*−1, *y*, *z*+1; (iii) *x*−1/2, −*y*+3/2, *z*+1/2; (iv) −*x*, −*y*+1, −*z*+1; (v) *x*+1/2, −*y*+3/2, *z*−1/2; (vi) −*x*+1/2, *y*+1/2, −*z*+1/2; (vii) *x*−1/2, −*y*+3/2, *z*−1/2; (viii) −*x*, −*y*+2, −*z*+1; (ix) *x*, *y*, *z*−1; (x) *x*−1, *y*, *z*.
